# Supplementary material for: Long-Term Adoption or Abandonment of Smart Technology in the Chinese Elderly Home Care Environment: A Qualitative Research Study
Source: Healthcare (Basel). 2023 Aug 31;11(17):2440. doi: 10.3390/healthcare11172440 (PMC10486740; doi:10.3390/healthcare11172440)
Supplement: Supplementary file 1 [file healthcare-11-02440-s001.zip › healthcare-2566839-supplementary.pdf]

Supplementary Interview Outline

Note：

The question numbered A is for the elderly who are using smart technology for a long time in the home care environment.

The question numbered B is for the elderly who have abandoned using smart technology.

Main problems：

1. a.b. How do you understand the smart technology in the home care environment? How did you learn about it?
2. a. What smart technologies do you use in the home care environment?
   b. What smart technologies have you ever used in a home care setting?
3. a.b. What do you think are the advantages (or disadvantages) of smart technology compared with traditional service ways?
(Traditional service means providing nursing services by family members or going directly to hospitals, institutions and other services without the intervention of modern technology)
4. a.b. Do you think smart technology is convenient for you to use? Or have you ever had any trouble using smart technology? Please introduce your experience in detail.
5. a.b. Do you think smart technology can be used for a long time for you? Please introduce your experience in detail.
6. a.b. Do you have any concerns when using smart technology? Please introduce your views in detail.
7. a. What causes you to use smart technology for a long time?
   b. What caused you to abandon the use of smart technology?
8. a. What situations might you abandon the use of smart technology if they occur in the future?
   b. What situations might you use smart technology again if they occur in the future?
9. What do you think the smart technology in the home care environment needs to

be improved?

General problems：

(1) Can you explain this in detail?

(2) Can you think of an example?

(3) How do you feel about it?

(4) What do you suggest?

(5) Do you have anything else to add?
